# Health-Promoting Properties of Lactobacilli in Fermented Dairy Products

**DOI:** 10.3389/fmicb.2021.673890

**Published:** 2021-05-21

**Authors:** Yantyati Widyastuti, Andi Febrisiantosa, Flavio Tidona

**Affiliations:** ^1^Research Center for Biotechnology, Indonesian Institute of Sciences (LIPI), Cibinong, Indonesia; ^2^Research Division for Natural Product Technology, Indonesian Institute of Sciences (LIPI), Yogyakarta, Indonesia; ^3^Council for Agricultural Research and Economics–Research Center for Animal Production and Aquaculture (CREA-ZA), Lodi, Italy

**Keywords:** lactobacilli, milk, fermentation, probiotics, post-biotics

## Abstract

Bacteria of the genus *Lactobacillus* have been employed in food fermentation for decades. Fermented dairy products, such as cheese and yogurt, are products of high value known as functional food and widely consumed due to their positive health impact. Fermentation was originally based on conversion of carbohydrate into organic acids, mostly lactic acid, intended to preserve nutrient in milk, but then it develops in other disclosure of capabilities associates with health benefit. It is expected that during the manufacture of fermented dairy products, some bioactive peptides from milk protein are released through proteolysis. Lactobacilli have been recognized and received increasing attention as probiotics by balancing gut microbial population. Information of molecular mechanisms of genome sequence focusing on the microbial that normally inhabit gut may explain as to how these bacteria positively give impact on improving host health. Recent post-biotics concept revealed that health benefit can also be associated after bacterial lysis. This mini review focuses on the contribution of lactobacilli in dairy fermentation with health-promoting properties on human health.

## Introduction

Fermented foods represent a distinct food culture in every community in the world, symbolizing the heritage and socio-cultural aspects of the people (Tamang et al., 2016). The practice of consuming fermented foods has prevailed across civilizations and strata of societies over centuries because there is an obvious tangible benefit to the consumers of such products (Bagchi, 2014). Recent situation corroborates the statement regarding consumption of fermented food as healthy food which plays an important role in the maintenance of human health condition. Currently, this turned out to be a modern lifestyle.

Lactic acid bacteria, particularly genus *Lactobacillus*, have been involved and employed in food microbiology especially in the fermentation of milk due to their high potential to produce important metabolites and improve the quality of the product. Moreover, lactobacilli have been documented to have a long history of safe use, supported by recognition of Generally Recognized as Safe by the Food and Drug Administration or Qualified Presumption of Safety by European Food Safety Authority (Bernardeau et al., 2008). In addition, lactobacilli offer exciting research prospects for acquiring fundamental knowledge of how bacterial cells function in the gut ecosystem (Tannock, 2004). Taxonomy and phylogeni of the genus *Lactobacillus* have been recognized as rather complicated, because of great number of species with a diverse group of species originated from several nutrient-rich niches. Placement and grouping of existing species of *Lactobacillus* have been done based on the technology that has been developed such as reverse transcriptase sequencing of 16S rRNA (Collins et al., 1991) and genomics sequencing (Salvetti et al., 2012; Sun et al., 2015; Wittouck et al., 2019).

The health benefits of fermented foods may be expressed either directly through the interactions of ingested live microorganisms with the host, as probiotic effect, or indirectly as the result of the ingestion of microbial metabolites synthesized during fermentation as biogenic effect (Wilburn and Ryan, 2016). The effects of some fermented dairy products on human health may vary depending on the species and the fermentation processes. For instance, *Lactobacillus helveticus* can potentially affect human health through direct mechanisms such as the inhibition of pathogens, modification of gut microbiota and modulation of the host immune system (Taverniti and Guglielmetti, 2012). There are several effects on biological activities of *Lacticaseibacillus casei* strain Shirota as probiotics when consumed orally as live cells. Their modulating effect on intestinal function and other metabolites has been thought to be their primary benefit for human health mainly on improvement of intestinal function. Moreover, consumption of *L. casei* strain Shirota is very safe, not only for healthy subjects, but also for patients with a variety of diseases and conditions (Miyazaki and Matsuzaki, 2008). Health-promoting effects of fermented milk of *L. helveticus* IDCC3801 is shown through improvements of the cognitive function of the elderly people, as assessed by self-rating scales, cognitive tests, and biomarker analysis (Chung et al., 2014). While Jauhiainen et al. (2010) reported that long-term intervention with *L. helveticus* fermented milk reduces augmentation index in hypertensive subjects.

## Taxonomy and Characteristics of Lactobacilli

The genus *Lactobacillus* is classified in the phylum *Firmicutes*, class *Bacilli*, order *Lactobacillales*, and family *Lactobacillaceae*. Within the family *Lactobacillaceae*, genus *Lactobacillus* represents the largest and most diverse group as indicated by great number of its species, which reaches 261 species (Zheng et al., 2020). Additional of proposal of new species of this genus has increased rapidly especially in the last 20 years. Thus, reclassification of the genus *Lactobacillus* based on core genome phylogeny (conserved) pairwise average amino acid identity, clade-specific signature genes, physiological criteria and the ecology have been undertaken. Reclassification of the genus *Lactobacillus* into 25 genera including the emended genus *Lactobacillus* have been referred to as the *Lactobacillus delbrueckii* group, *Paralactobacillus*, and 23 novel genera for which the names *Holzapfelia, Amylolactobacillus, Bombilactobacillus, Companilactobacillus, Lapidilactobacillus, Agrilactobacillus, Schleiferilactobacillus, Loigolactobacilus, Lacticaseibacillus, Latilactobacillus, Dellaglioa, Liquorilactobacillus, Ligilactobacillus, Lactiplantibacillus, Furfurilactobacillus, Paucilactobacillus, Limosilactobacillus, Fructilactobacillus, Acetilactobacillus, Apilactobacillus, Levilactobacillus, Secundilactobacillus*, and *Lentilactobacillus* are proposed (Zheng et al., 2020). This new Lactobacillus taxonomic classification may facilitate our understanding of common mechanisms that could mediate probiotic health benefits. Division the genus *Lactobacillus* based on certain physiological and metabolic properties is timely that can anticipate the addition of new species in the near future.

Lactobacilli are characterized as Gram-positive bacteria with negative reaction to catalase, non-motile, and non-spore forming rods or coccobacilli often in chain configuration with their G+C content usually below 50 mol% (Hammes and Vogel, 1995). Based on the ability to ferment carbohydrate, they are divided into species of homofermentative and heterofermentative, that convert carbohydrate into lactic acid, and lactic acid, acetic acid, ethanol, and CO_2_, respectively. Lactose catabolism is the most relevant subject area of study related to metabolism in milk fermentation.

Recently, sequencing of whole bacterial genomes revealed important traits in characterization. Functional genomics approaches in lactobacilli, that are associated with food and health, rely on its complete genome sequence (Douillard and de Vos, 2014). It is also emphasized that genomic information allowed a better comprehension of lactobacilli features such as their physiology, metabolic capabilities, probiotic potential, key gene features, and niche adaptation. Regarding the safety of lactobacilli strains as probiotics, their assessment should be performed using the most updated methodology (Salvetti et al., 2012). Some lactobacilli have been reported as strains with high probiotics potential and support efforts to improve probiotics quality such as *Ligilactobacillus salivarius* strains BCRC14759 and BCRC 12574 with the highest exopolysaccharide (EPS) production (Chiu et al., 2017), *Lactobacillus johnsonii* ZLJ010 with better adaptation to the gut environment and its probiotic functionalities (Zhang et al., 2019), and *Lactobacillus helveticus* D75 and D76 that are able to inhibit the growth of pathogens and pathobionts (Toropov et al., 2020).

## Lactobacilli in Fermented Dairy Products

The basic fermentative process carried out by lactobacilli can already deliver dairy products with enhanced nutritional properties, as they are able to remove antinutritional factors (mainly lactose and galactose, preventing lactose intolerance, or galactosemia), increase the digestibility and the biological value of the proteins. Lactobacilli strains generally employed as starter cultures show optimal growths in milk although they may display limited biosynthetic abilities compared to wild strains (Settanni and Moschetti, 2010). This is the reason why dairy LAB are defined as “domesticated” strains, revealing the loss of ancestral genes and metabolic simplification toward adaptation to a given environment, as confirmed by comparative genome analysis of multiple species (McAuliffe, 2018).

Notwithstanding, lactobacilli harbor a huge phenotypic and genetic diversity, that is extensively investigated to select strains exhibiting traits of interest. Commercial starter cultures are able to deeply influence the flavor and texture of the product through the breakdown of milk proteins, fats, and other milk constituents (Giraffa et al., 2010). Conversely, lactobacilli can also improve the quality characteristics of the fermented products through specific biosynthetic capabilities, such as the synthesis of thickening hydrocolloids (exopolysaccharides) that enable to deliver yogurts with ameliorated rheological properties (Torino et al., 2015). However, the largest success of lactobacilli in fermented dairy products is assigned to probiotics, that may responsible of the fermentation process together with associated starter cultures or added as adjunct cultures. Ancient people in Asia were proficient in practicing or making fermented milk in a simple way and nowadays they are still produced under the name of traditional fermented milks. The reason of such practice can be due to limited facilities and possibility to use pure starter cultures. Fermentation in these products is generally driven by unidentified indigenous microorganisms derived from the residual milk of previous day. Fermented milk products of Asia including Dahi, a yogurt-like from India, or Dadih from Indonesia, Kumys, originated from Central Asia and Tarag or Airag, a hard-type yogurt from inner Mongolia. Among the mix microorganisms present in Kumys there are *Lactobacillus delbrueckii* subsp. *bulgaricus, Lactobacillus kefiranofaciens, Lactobacillus acidophilus, Lactococcus lactis* and yeasts, such as *Candida* spp., *Kluyveromyces lactis*, and *Torula* spp. Tarag is considered to be a rich source of vitamins and minerals (Akuzawa et al., 2011). Whereas, *L. helveticus* and *L. kefiranofaciens* were isolated from Airag and *L. delbrueckii* subsp. *bulgaricus, L. helveticus*, and *Streptococcus thermophilus* were the predominant isolates from Tarag (Watanabe et al., 2008). Meanwhile, Yakult and Calpis were developed in Japan. Yakult is well-known fermented milk product on account of various claims of its health-promoting properties. The strain used in Yakult manufacture, *L. casei* strain Shirota (reclassified as *Lacticaseibacillus paracasei* subsp. *paracasei*), is an indigenous human intestinal bacterium, able to resist gastric juices and bile. Calpis, which originated from the fermented milk of Mongolian nomads, contains several peptides derived from milk proteins, claiming a physiological effect on lowering of blood pressure in hypertensive human subjects (Akuzawa et al., 2011). The ability of specific indigenous lactobacilli enabled to develop successful industrial products from traditional fermented milks. In Europe, fermented milks were traditionally prepared and consumed, especially from northern Scandinavia. The main characteristic of these products is the slimy texture of the milks caused by presence of ropy exopolysaccharides and today commercial products based on old starter cultures are manufactured such as “Tjukkmjølk” in Norway, “Långfil” in Sweden, “Viili” in Finland, and “Skyr” in Iceland (Fondén et al., 2006). In eastern Europe and Mediterranean area (ancient Greece and Rome) the production of low alcoholic fermented drinks were used both for nourishment and medicines to relieve pain and to prevent or treat diseases (Marshall and Mejia, 2011). Koumiss, a traditional alcoholic fermented beverage of Kazakh nomads made from mares' milk had been used by Russian doctors for the treatment of tuberculosis and diarrhea (Baschali et al., 2017). Only many years later the presence of lactobacilli with probiotic properties was demonstrated.

Main species identified with probiotic properties belong to *Lacticaseibacillus rhamnosus Lactobacillus acidophilus and Lacticaseibacillus casei and Lactiplantibacillus plantarum* that are generally found in yogurts, kefir and other fermented milks (Table 1). The efficacy of probiotic lactobacilli was also evaluated in cheeses, although the survival of cells through processing and aging may be more challenging, providing positive mucosal immune responses *in vivo*, in a mice model (Medici et al., 2004). There are also various application of Lactobacilli strains in the dairy products that have been developed for human health therapy as reported in Table 1.

**Table 1:** Beneficial effect of probiotic dairy products to human health.

**Product**	**Lactic acid bacteria strain**	**Functional benefit to human**	**References**
**Europe**			
Yohurt-Like beverage	*L. rhamnosus* SP1, *W. confusa* DSM 20194, *L. plantarum* T6B10	Free amino acids and γ-Aminobutyric acid (GABA), polyphenols availability, antioxidant activity (up to 54%), and protein digestibility	Lorusso et al., 2018
Synbiotic yohurt	*L. acidophilus, B. longum*	Increase in HDL-cholesterol and improved LDL/HDL ratio	Kiessling et al., 2002
Probiotic fresh cheese	*L. acidhophilus A9,* *B. bifidum A12*, *and* *L. paracasei A13*	Immunomodulating capacity	Medici et al., 2004
Edam cheese	*L. rhamnosus* LC705, *L. rhamnosus* GG ATCC*53103 (*LGG*)*	Decrease oral cavity risk by 21%	Ahola et al., 2002
Capsule containing probiotics	*L plantarum* P17630	Suppress *Candida albicans* of patients with vulvovaginal candidiasis (VVC)	Seta et al., 2014
Supplement	*L. casei* Shirota	Improve immune status of athletes	Gleeson et al., 2016
Supplement	*L. subtilis, S. faecium*	Restoration of bowel flora and improvement of lipopolysaccharide in patients with alcoholic hepatitis	Han et al., 2015
**Asia**			
Iranian traditional cheese	*L. plantarum*	Stomach ulcer	Nasrabadi et al., 2011
Iranian probiotic yogurt	*L. brevis*	Wound, diabetes type II	Ejtahed et al., 2011
Probiotic yogurt	*L. delbrueckii* subsp. *bulgaricus* and *S.thermophilus*. *B. animalis* subsp. *lactis* Bb12 (DSM 10140) and *L. acidophilus* strain La5	Decrease in HbA1c and TNF-α levels	Mohamadshahi et al., 2014
Probiotic supplement	*B. bifidum, L. casei*, *L. acidophilus 2*	Glycemic control, HDL-cholesterol, total-/HDL-cholesterol ratio, biomarkers of inflammation, and oxidative stress in diabetic patients with CHD	Raygan et al., 2018
Yak milk	*L. casei* SB27	Inhibit the proliferation of HT-29 colorectal cancer cells and up-regulated the 7expressions of Bad, Bax, Caspase-3, and−8 genes	Di et al., 2017
Tibetan kefir	*L. plantarum* YW11	Increased the content of short-chain fatty acids, antioxidant, and gut microbiota regulating activities	Zhang et al., 2017
Fermented milk	*L. delbrueckii* ssp. *bulgaricus* SRFM-1	Antioxidant	Tang et al., 2017
Fermented milk	*L. rhamnosus* SD11	Oral health by reducing salivary levels mutans streptococci	Rungsri et al., 2017
Iranian traditional cheese	*L. plantarum*	Enhanced gastric ulcer healing via stimulating immune system and fibroblast increasing	Nasrabadi et al., 2011
Kurut (Tibet traditional fermented milk)	*L helveticus* H9	Antihypertensive	Chen et al., 2014
Traditional fermented yak's milk (Tibet, China)	*L. coryniformis* subsp *torquens* T3L	Immunomodulating effect	Tuo et al., 2011
Kefir (Tibet, Kaukasian mountain, China)	*L. kefiranofaciens*	Antibacterial, hypocholesterolemic, antihypertensive, anti-inflammatory, anti-diabetic, antioxidant, anti-carcinogenic, anti-allergenic activities	Rosa et al., 2017
Chinese sauerkraut	*L. plantarum* S4-1	Reduce serum cholesterol level	Yu et al., 2013
Xueo (traditional fermented yak milk), China	*L. fermentum SCA52*	Antimicrobial activity	Ao et al., 2012
Fermented milk (Kefir) Japan	*Lactobacillus/Lactococcus* sp.	Anti-Obesity, and anti-non-alcoholic fatty liver disease activity	Kim et al., 2017
Fermented milk product (Japan)	*L. helveticus* (probiotics)	Improved the cognitive function of healthy adults	Ohsawa et al., 2018
Koumiss (China)	*L. paracasei* supsp. *paracasei* M5L	Immunomodulating effect	Tuo et al., 2011
Fermented Milk (China)	*L. paracasei* Jlus66	Protecting from Nonalcoholic fatty liver disease (NAFLD)	Ye et al., 2017
Fermented milk enriched with gamma-Aminobutyric acid (China)	*L. brevis* and *L. plantarum*	Promote relaxation and reduce anxiety	Yu et al., 2020
Dadih (Fermented Buffalo milk) Indonesia	*L. plantarum* IS-10506	Therapy of children with atopic dermatitis	Prakoeswa et al., 2017
Dahi (Yogurt like) India Pakistan	*L. acidophilus* LA 02, *L. delbrueckii* subsp. bulgaricus	Reduce functional constipation, prevention of recurrence of vulvovaginal candidiasis	Mitelmão et al., 2021
Koumiss (Mare's milk) Central Asia	*L. fermentum* SM-7	Cholesterol-Lowering ability for hypercholesterolemia treatment	Thumu and Halami, 2020
Yakult	*L. casei Shirota* and *B. breve* Yakult	Improve symptoms and decrease hydrogen production intake in lactose-intolerant patients	Almeida et al., 2012
**America**			
Milk Kefir	*L. kefiri*	Down-Regulate expression of pro-inflammatory mediators and increase anti-inflammatory molecules in the gut immune system	Rosa et al., 2017
Fermented milk (yogurt)	*L. helveticus* R389	Delayed breast tumor growth by decreasing IL-6 and increasing IL-10 in serum	Leblanc and De Perdigo, 2010
Fermented milk	*L. rhamnosus* GG and *S. boulardii*	To prevent antibiotic-associated diarrhea and to treat acute infectious diarrhea	Barnes and Yeh, 2015
Fermented milk	*L. acidophilus* and *L. casei*	Prevention of antibiotic-associated diarrhea	Beausoleil et al., 2007
Fermented milk (Kefir) Argentina	*L. paracasei* CIDCA8339	Antiinflamatory effect	Bengoa et al., 2019
**Africa**			
Yogurt	*L. rhamnosus* GR-1 and *L. reuteri* RC-14	Increased CD4 cell count, resolved diarrhea, flatulence, and nausea of HIV/AIDS patients	Anukam et al., 2008
Fermented milk product (*lait caillé*) from Northern Senegal	*L. rhamnosus* yoba 2012	Health-Promoting probiotic, prevalence of malnutrition	Parker et al., 2018
Ghanaian traditionally fermented milk	*E. faecium, L. fermentum, L. plantarum, and P. acidilactici*	Antimicrobial pathogent/Antibiotic activity	Motey et al., 2021

## Fermented Dairy Products as Functional Food

Lactobacilli are able to produce an array of metabolites that may be useful to enrich the fermented food, increasing the health-associated value. Among the biologically active metabolites produced after fermentation, a major role is played by bioactive peptides, that are encrypted within milk proteins and made available by the proteolytic system of lactobacilli. Milk proteins are currently considered a relevant source of bioactive peptides but only the strain that possess specific cell envelope proteases and peptidases are able to release oligopeptides or peptides that exert a specific activity, based on the amino acid composition and sequence (Hafeez et al., 2014). The demonstrated presence of such peptides in a fermented product enables to define the category of functional foods, which provide health benefits beyond its nutritional value. One of the first functional dairy food was a soft drink fermented by *Lactobacillus helveticus* CP790 and a strain of *Saccharomyces cerevisiae* with antihypertensive effects, although several other products, each with a specific health claim, have been launched into the market (Tidona et al., 2009). These peptides are generally tasteless but those containing hydrophobic amino acids may result bitter, thus it also has to be taken into account. Some rare strains of lactobacilli produce inulin, a prebiotic that is also reported to improve the quality of skim milk fermented by *L. acidophilus, L. rhamnosus, Lactobacillus bulgaricus* (Patel and Goyal, 2012). A functionalized yogurt was developed using selected strains of *L. bulgaricus* (associated to *S. thermophilus)* which naturally increased the folate concentration by almost 250% (Laiño et al., 2013). Besides peptides and vitamins, many other bioactive metabolites can also be synthetized during fermentation, such as bacteriocins and enzymes that could be exploited to produce natural food ingredients. Functional oligosaccharides (GOS and FOS) have been found effective in the prevention of dental caries, facilitation of mineral absorption, antioxidant properties, enhancement of immunity and alternative regulators of blood glucose in diabetics (Patel and Goyal, 2011). The ability of a specifically selected strains may be addressed to provide an added-value to fermented products or to diversify product range, which is the key to implement successful strategies. In other cases, the presence of virtuous lactobacilli is associated to health-promoting properties, such as immune-stimulatory antitumoral effects (Nishimura, 2014) or to lower cholesterol level (Jones et al., 2012). Among fermented milks, Kefir can be naturally considered a functional food given the numerous reports claiming for positive effects, i.e., anti-allergenic, cholesterol metabolism, angiotensin converting enzyme (ACE) inhibition, wound healing, anti-carcinogenic, antimicrobial and gastrointestinal health (Bourrie et al., 2016), although its microbial composition is rich of different LAB species and yeasts.

## Metabolism in Lactobacilli: from Probiotics to Post-biotics

The effectiveness of probiotics is related to specific strains, a minimum dosage of viable cells and the specific health claim, according to the World Gastroenterology Organization guidelines. When probiotics are considered dietary supplements, there should be sound scientific evidences for the use of a strain and the beneficial effect claimed; analogously, for probiotic drugs, clinical trials for a specific treatment or disease associated to the presence of a strain should be demonstrated, in compliance with drug legislation (García-Burgos et al., 2020). One of the most common use of probiotics is defined by the cure of *Helicobacter pylori* infection with Lactobacilli, even in pediatric age, prevention of allergies, treatment of chronic disease irritable bowel syndrome, treatment of inflammatory bowel disease, treatment of vaginitis, and generally suggested after the use of antibiotics (McFarland, 2015). The global market of probiotics is still the largest in the section of functional food and it comprises a huge variety of food products where probiotics are added, mainly to dairy products (yogurts, fermented milks, ice creams, and cheeses), even if the application involving non-dairy matrices (plant-based fermented products, juices, and vegan products) are rapidly emerging. The main role of probiotics is the ability to modulate the intestinal microbiota, although this complex mechanism is still poorly understood. However, it was observed how probiotic lactobacilli promote the growth of beneficial bacteria against harmful microorganisms, such as *Echerichia coli, Listeria monocytogenes, Staphylococcus aureus, Salmonella typhimurium, Salmonella enteritidis, Shigella flexneri, Pseudomonas aeruginosa*, and *Yersinia* enterocolitica (Bourrie et al., 2016). In commercial applications, when viability of probiotic cultures is concerned, the beneficial effect of the product may be compromised. In addition to this drawback, starter cultures combined to probiotics in fermented products can possess antibiotic resistance genes that may be potentially transferred to pathogen microorganisms (Hummel et al., 2007). Therefore, current research has also investigated the efficacy of the microbial metabolism of probiotic lactobacilli, devoid of the live cells. The term paraprobiotics, also called inactivated probiotics or ghost probiotics, was introduced to refer to non-viable microbial cells or to raw cellular extracts which, administered orally or topically in adequate amounts, are able to confer health benefits (Taverniti and Guglielmetti, 2011). The concept of post-biotics can also be extended to bioactive metabolites or their cell-free supernatants, that are considered a safe and effective alternative, in comparison to live bacteria, to maintain gut health and to prevent inflammatory bowel diseases. Several strains of *L. plantarum* are reported to exhibit post-biotic effects, such as those producing bacteriocins employed as antibiotic replacers in the feed of poultry and rats (Foo et al., 2003; Thanh et al., 2009). Post-biotics from *L. plantarum* also showed cytotoxic effects and induction of apoptosis against malignant cancer and they were proposed as supplement or adjunctive treatment for cancer (Chuah et al., 2019). There are already post-biotics applications in pharmaceutical products, which were legally approved with success, both for medical, veterinary, and food purposes. Several strains (*L. acidophilus, L. plantarum, L. rhamnosus, L. bulgaricus, L. salivarius, L. casei, L. reuteri, L. sporogenes, B. bifidum*) are commercialized as immunomodulatory supplements. The bioactive metabolites commonly found in post-biotics can be related to organic acids, short-chain fatty acids, carbohydrates, antimicrobial peptides, enzymes, vitamins, cofactors, immune-signaling compounds, and complex agents (Moradi et al., 2019). Post-biotic products are generally more convenient than probiotics due to industrial advantages, as they are stable in a wide range of pH and temperature, often do not require cold-chain, extended shelf-life and little interaction with the matrices (Barros et al., 2020). Furthermore, post-biotics were applied in food active packaging, especially through the incorporation of bacteriocins produced by LAB in antimicrobial films. For instance, the strain of *L. lactis* ATCC 11454 producing nisin was incorporated in active films based on alginate and collagen, showing a strong inhibition vs. *List. monocytogenes, Staph. aureus*, and *E. coli*. (Ma et al., 2020).

## Conclusion

Lactobacilli, that hold a long history of safe use, are still of great importance for the dairy fermentations. With the recent and growing information on their genome sequences, the role of lactobacilli in fermentation can be optimized and tailored, giving insights into new product development. The production of bioactive peptides by lactobacilli, although limited to a few strains, represent the most important driver to produce functional foods with specific health claims. The pharmaceutical biotechnology based on the use of lactobacilli with probiotics properties, are highly supported by the evidence of health benefits to the host. On the other hand, post-biotics from lactobacilli, which refer to non-viable microbial cells or their raw cellular extracts are also important emerging alternatives with health-promoting effects.

## Author Contributions

YW, AF, and FT reviewed the literature and wrote the manuscript. AF summarized and prepared the table. All authors approved the submitted version.

## Conflict of Interest

The authors declare that the research was conducted in the absence of any commercial or financial relationships that could be construed as a potential conflict of interest.
